# In this month’s Bulletin

**DOI:** 10.2471/BLT.21.000421

**Published:** 2021-04-01

In the editorial section, Shelly Chadha et al. (242) introduce WHO’s first world report on hearing. Viroj Tangcharoensathien et al. (243) call for submissions to a theme issue on effective policy responses during the COVID-19 pandemic.

In the news section, Andréia Azevedo Soares (246–247) reports on initiatives aiming to improve access to insulin. Yemurai Machirori talks to Gary Humphreys (248–249) about advocating for attention to the needs of people with type 1 diabetes.

## European region

### Eliminating viral hepatitis 

Jin Un Kim et al. (280–286) argue that migrant populations need better access to diagnosis and treatment.

## New Zealand

### Damp, mould, cold and injury

Lynn Riggs et al. (259–270) quantify disease caused by substandard housing.

## Pakistan 

### Infant male circumcision 

Shazia Moosa et al. (250–258) study programme implementation.

## Sierra Leone 

### Safe and dignified burials 

Padraig Lyons et al. (271–279) examine what worked during the 2014-2016 Ebola outbreak.

## South-East Asia Region

### Control of syphilis, gonorrhea, chlamydia and chancroid 

Mukta Sharma et al. (304–311) review progress towards elimination targets for sexually transmittable diseases.

## Sub-Saharan Africa 

### Human immunodeficiency virus, syphilis and hepatitis B 

Jennifer Cohn et al. (287–295) present the case for triple elimination of mother-to-child transmission.

## Thailand

### Public health policies in a pandemic

Natthaprang Nittayasoot et al. (312–318) explore elements of the COVID-19 response.

## Global

### Improving medical education

Tommy Hana et al. (296–303) examine the coverage of transgender health.

### Providing essential medicines

Christine Årdal et al. (319–320) argue for supply chain transparency.

